# The stability of blood eosinophils in stable chronic obstructive pulmonary disease: a retrospective study in Belgian primary care

**DOI:** 10.1186/s12890-020-01234-3

**Published:** 2020-07-22

**Authors:** Inès Van Rossem, Jan Vandevoorde, Shane Hanon, Sander Deridder, Eef Vanderhelst

**Affiliations:** 1 Department of Family Medicine and Chronic Care, Vrije Universiteit Brussel, Laarbeeklaan 103, B-1090 Brussels, Belgium; 2 Respiratory Division, Universitair Ziekenhuis Brussel, Laarbeeklaan 101, B-1090 Brussels, Belgium; 3 Department of Chemical Engineering, Process and Environmental Technology Lab, Katholieke Universiteit Leuven, Jan Pieter de Nayerlaan 5, 2860 Sint-Katelijne-Waver, Belgium

**Keywords:** COPD, Eosinophils, Biomarkers, Stability/reproducibility, Primary care, Airway inflammation

## Abstract

**Background:**

Blood eosinophil counts (BEC) were recently included in the 2019 Global Initiative for Obstructive Lung Disease (GOLD) guideline as an easily accessible theragnostic biomarker for Chronic Obstructive Pulmonary Disease (COPD). However, the stability of BEC remains insufficiently studied.

**Methods:**

We conducted a retrospective study in six primary care practices in Belgium on data from Electronic Health Records of stable COPD patients, to characterise the stability of blood eosinophils over time. We report the percentage of patients with BEC persistently below or above the 2019 GOLD guideline thresholds (100 and 300 cells/μL). For each patient the mean, standard deviation (SD) and relative standard deviation (RSD) of the BEC were calculated to determine the intra-patient variability.

**Results:**

Ninety-eight patients were included, yielding 1082 eosinophil measurements (median 8 measurements/patient), with BEC ranging between 0 and 1504 cells/μL. Four (4.1%) patients had BEC persistently below 100 cells/μL, 34 (34.7%) had measurements persistently above this threshold. Approximately half of the patients (51.0%) had BEC persistently below 300 cells/μL and 3 (3.1%) patients had counts persistently above this threshold. 28.6% of patients crossed both threshold values throughout the registration period. The mean BEC per patient ranged between 15 and 846 cells/μL with an intra-patient SD between 5 and 658 cells/μL. The mean intra-patient RSD was 0.46. There was a significant strong positive correlation (Pearson analyses) between the mean BEC and SD (*r* = 0.765; *n* = 98). Simple linear regression was used to further describe the influence of the mean eosinophil count on the SD (B = 0.500; 95%CI 0.415–0.586; *n* = 98; *p* < 0.001).

**Conclusion:**

BEC can be variable in individual COPD patients. Therefore, the use of a single measurement to guide therapeutic decisions remains debatable. Further prospective research remains necessary to validate the reproducibility of this biomarker.

## Background

Chronic Obstructive Pulmonary Disease (COPD) is a common, preventable and treatable disease that is characterised by persistent respiratory symptoms and airflow limitation [1]. This highly prevalent chronic disease is often also managed in primary care and is associated with high morbidity and mortality. It is a heterogeneous disease with several clinical and pathobiological characteristics such as emphysema, small airway disorders and chronic airway inflammation [1]. In the past, the inflammation related to COPD was often considered to be predominantly neutrophilic, but there is growing evidence that it can also be of eosinophilic nature [2–4]. Blood eosinophils have been suggested as a biomarker for this eosinophilic inflammation based on the possible association between blood and lung eosinophil counts [5, 6]. This has led to the inclusion of Blood Eosinophil Counts (BEC) in the Global Initiative for Obstructive Lung Disease (GOLD) report [1]. In this guideline different thresholds (100 and 300 cells/μL) for BEC are proposed to determine the eligibility for maintenance treatment with inhaled corticosteroids (ICS) in exacerbating or GOLD group D COPD patients [1].

Given their accessibility and the link with response to ICS [7, 8], newly determined or historical BEC can indeed be an interesting tool to guide clinical decisions. However, a good biomarker should by definition be reproducible [9], and the stability of BEC and its influencing factors, remain insufficiently studied [10]. If BEC are indeed variable over time, causing a threshold to be crossed, patients could be assigned to different treatment categories depending on the time of measurement.

The current paper reports on real world data from primary care, determining the intra-patient variability of BEC over time in stable COPD patients. In addition, the relationship between patient characteristics and the patients mean eosinophil count and eosinophil variability was evaluated.

## Methods

The aim of this study was to determine the intra-patient variability of BEC over time in COPD patients in Belgium. Approval of the study by the local medical ethics committee (UZ Brussel, BUN 143201837046) was obtained. All data, ranging from 1996 to 2019, were gathered from the Electronic Health Record (EHR) of spirometry proven COPD patients from six primary care practices in Belgium. Patients with concurrent asthma or less than two BEC were excluded. All retrieved BEC measured in stable state were included for analysis. Exclusion of BEC measured during an exacerbation was done by searching each patients’ file for records of hospitalisation for an exacerbation, records of exacerbations without hospitalisation and the prescription of oral corticosteroids and/or antibiotics in combination with respiratory symptoms. All BEC in a one-month interval around these dates were excluded.

We reported the percentage of patients with BEC persistently below the value of 100 cells/μL and persistently above the value of 300 cells/μL, as well as the proportion of patients crossing both thresholds (i.e. presenting at times BEC below 100 cells/μL and at times BEC above 300 cells/μL). For each patient mean BEC, standard deviation (SD) and relative standard deviation (RSD = SD/mean) were calculated to characterise the intra-patient variability. The effect of patient characteristics (see Table 1) on the mean eosinophil count and SD was analysed by Pearson/Spearman correlation (two-tailed) and linear regression. Comorbidities were defined in accordance with the 2018 GOLD guideline [11]. Statistical analysis was performed using IBM SPSS statistics 25 (SPSS 25.0; IBM, Armonk, NY, USA).

**Table 1:** Patient characteristics of the study population

Total population, n (%)	98 (100.0)
Caucasian, n (%)	98 (100.0)
Male, n (%)	73 (74.5)
Smoking status	
Smokers, n (%)	38 (38.8)
Ex-smokers, n (%)	54 (55.1)
Unknown, n (%)	6 (6.1)
Age, mean (SD), years	71.1 (10.3)
FEV_1_% predicted, mean (SD)^a^	60.1 (18.2)
Spirometric GOLD grade, n (%)	
Grade 1	20 (20.4)
Grade 2	51 (52.0)
Grade 3	22 (22.4)
Grade 4	5 (5.1)
BMI, mean (SD), kg/m^2^	24.9 (5.7)
Patients with at least one exacerbation, n (%)	58 (59.2)
Patients with at least one severe exacerbation, n (%)	29 (29.6)
Comorbidities, n (%)	132 (100.0)
Hypertension	28 (21.2)
Gastroesophageal reflux	21 (15.9)
Arrhythmia	19 (14.4)
Depression	14 (10.6)
Ischaemic heart disease	12 (9.1)
Metabolic syndrome and diabetes	10 (7.6)
Peripheral vascular disease	10 (7.6)
Anxiety	4 (3.0)
Osteoporosis	4 (3.0)
Obstructive sleep apnea	3 (2.3)
Bronchiectasis	1 (0.8)
Lung cancer	0 (0.0)

*SD* Standard Deviation, *FEV*_*1*_ Forced Expiratory Volume in 1 s, *BMI* Body Mass Index

^a^Post bronchodilation

## Results

In this study 6 primary care practices employing 15 full time equivalent GP’s participated. In our cohort (Table 1) 98 COPD patients (all Caucasian, 73 male) were included with a mean age of 71.1 ± 10.3 years, a mean Forced Expiratory Volume in 1 s of 60.1 ± 18.2% predicted and a mean Body Mass Index of 25 ± 5.7 kg/m^2^. Thirty-eight patients were current smokers (38.8%), for 6 patients no registration of their smoking habits was found in the EHR (6.1%). For 58 (59.2%) patients at least one exacerbation regardless of severity, and for 29 (29.6%) at least one severe exacerbation were recorded in the patient files. The mean number of comorbidities per patient was 1.3, arterial hypertension being the most frequent.

**Fig. 1:**
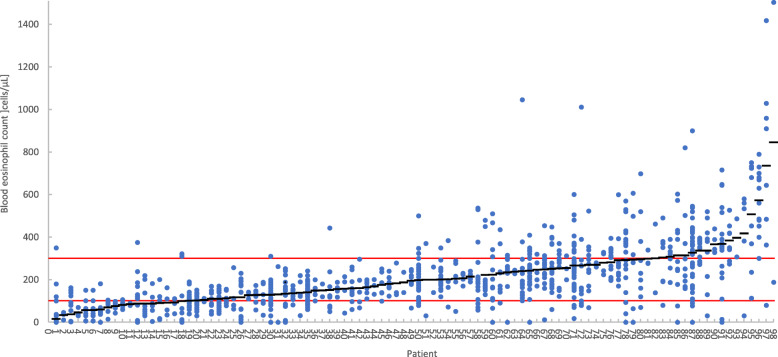
Repeated measures of blood eosinophils (blue dots) per patient in a primary care COPD cohort (*n* = 98). Legend: Patients are ordered according to increasing mean blood eosinophils count (black dashes). The thresholds of 100 and 300 cells/μL are indicated (red lines)

A total of 1082 BEC were recorded, ranging between 0 and 1504 cells/μL, resulting in a mean (median) of 11.0 (8.0) measurements per patient (Fig. 1). Four (4.1%) patients had BEC persistently below 100 cells/μL, 34 (34.7%) had measurements persistently above this threshold. Approximately half of the patients (51.0%) had BEC persistently below 300 cells/μL and 3 (3.1%) patients had counts persistently above this threshold. In total 28 (28.6%) patients crossed both thresholds throughout the registration period. The mean eosinophil count per patient ranged between 15 and 846 cells/μL (Fig. 1) with a SD between 5 and 658 cells/μL (mean SD = 89 cells/μL). The mean RSD was 0.46. There was a significant and strong positive correlation between mean eosinophil count and SD (*r* = 0.765; *n* = 98; *p* < 0.001). Simple linear regression was used to further describe the influence of the mean eosinophil count on the SD (B = 0.500; 95%CI 0.415–0.586; *n* = 98; *p* < 0.001). The correlation analyses between patient characteristics and mean eosinophil count or SD, showed only a significant but weak positive correlation between mean eosinophil count and age (*r* = 0.227; *n* = 98; *p* = 0.025) and between exacerbation and SD (≥1 exacerbation regardless of severity *r* = 0.210; *n* = 98; *p* = 0.038). The correlation with SD was also found when severe exacerbations only were taken into account (≥1 severe exacerbation *r* = 0.249; *n* = 98; *p* = 0.013). Therefore, further multiple linear regression analyses were not performed.

## Discussion

As shown, our primary care cohort presented a large range of BEC over time. Moreover, the large SD’s (and RSD’s) could suggest a significant intra-patient variability over time, resulting in a considerable proportion of patients crossing over GOLD proposed threshold values when measured at different points in time. Previous studies confirm this variability of BEC [12, 13], others [14] report a higher stability. The strong correlation (*r* = 0.765) between mean eosinophil count and SD observed here, suggests a higher variability of blood eosinophils at higher levels, which is in line with previous findings [10, 14–16].

The positive correlation between age and mean eosinophil count found in this study is in line with the findings in the ECLIPSE study [4]. Some evidence also suggest an influence of sex on BEC [4] and BEC stability [10], but this could not be confirmed by our data. Concerning exacerbations, literature is somewhat contentious about the capability of BEC to predict future exacerbations [4, 8] possibly due to differences in composition of study populations (exacerbators vs. non exacerbators). Our analysis does not demonstrate a correlation between exacerbations and BEC. However, a significant but weak positive correlation was found between exacerbations and SD indicating that patients presenting exacerbations showed higher BEC variability. In our cohort, none of the other patient characteristics considered, showed significant correlation with the mean BEC or SD.

As previously argued, the data from the current study could indicate that using one single BEC measurement has limited value in guiding treatment choices. The recent study by Long et al. [15] however, reports that most of the patients remain in the same BEC category after 1 year, providing reassurance regarding therapeutic decisions based on BEC. On the other hand, our data shows that in 28.6% of the patients, the BEC were not ideal to guide the maintenance treatment, as BEC crossed both thresholds over time. The use of mean BEC of repeated measurements as a possible solution to overcome variability, as proposed in literature [15, 17], could be promising but needs further validation.

This real-world study on primary care data, for which there is more and more demand in respiratory medicine [18], is the first observational study in Belgium with such a large registration period and reporting such high numbers of BEC in total and per patient. Six primary care practices employing 15 full time equivalent GP’s participated. With an average in Belgium of 950 patients per GP [19] and an overall prevalence of COPD in primary care of 2.2% [20], a total of 98 patients meeting the stringent inclusion criteria is within expectations. As in real life, eosinophil measurements were performed by different laboratories at different time intervals, but their variability exceeded the reported variability of the laboratory analysis itself [21] and although asthma cannot be fully excluded, all COPD diagnoses were spirometry based. The effect of parasitic infections on BEC was not considered, but a large influence seems very unlikely in a study performed in Belgium. Of course, the retrospective nature of the study is a limitation, eg. the partitioning of patients in exacerbation groups. Also, our analysis was not limited to those COPD patients for whom ICS might be most relevant (exacerbating or GOLD group D patients). However, in our opinion, this cohort is representative of the real-world COPD population in primary care.

## Conclusion

We report large (R) SD values for BEC in individual patients. Consequently, a considerable proportion of the patients in this primary care cohort is crossing over threshold values over time. Therefore, the use of a single blood eosinophil measurement to guide therapeutic decisions remains debatable, making further prospective research necessary to validate the reproducibility of this biomarker.

## Data Availability

The datasets used and/or analysed during the current study are available from the corresponding author on reasonable request.

## Abbreviations

COPD: Chronic Obstructive Pulmonary Disease
BEC: Blood eosinophil counts
GOLD: Global Initiative for Obstructive Lung Disease
ICS: Inhaled corticosteroids
EHR: Electronic Health Record
SD: Standard deviation
RSD: Relative standard deviation
GP: General Practitioner
